# Executive Function and young children's Cardinality Principle: the mediating role of the Approximate Number System and the moderating role of age

**DOI:** 10.3389/fpsyg.2024.1495489

**Published:** 2024-11-13

**Authors:** Huanhuan Li, Huijuan Di, Bingyu Duan, Mengzhen Luo, Yifang Wang, Zhenglu Wang

**Affiliations:** ^1^College of Educational Science, Xinjiang Normal University, Urumqi, China; ^2^Department of Preschool Education, Hebei Normal University, Shijiazhuang, China; ^3^Shanghai Institute of Early Childhood Education, Shanghai Normal University, Shanghai, China

**Keywords:** Executive Function, Cardinality Principle, Approximate Number System, mediating role, moderating role

## Abstract

**Background:**

Executive Function and the Approximate Number System are well-established as critical components in developing the Cardinality Principle in young children. However, most existing studies explore the relationship between these variables in isolation without examining whether Approximate Number System mediates the relationship between Executive Function and the Cardinality Principle and the role of age in this. This study aimed to address this gap by investigating the mediating role of the Approximate Number System in the relationship between Executive Function and the Cardinality Principle and the moderating role of age in young children.

**Methods:**

This cross-sectional study was conducted in China from February to June 2024. A total of 203 young children (97 boys and 106 girls, Mean age = 68.93 ± 7.076 months) participated. Participants were assessed using a range of tests: the Day-Night Stroop Task, Digit Recall Task, Dimensional Change Card Sort Task, Panamath Test Software, How Many Task, and Give-N Task to measure Executive Function, Approximate Number System, and Cardinality Principle. Data were analyzed using SPSS 26.0 and PROCESS v4.1 (Model 4) to explore the relationships among Executive Function, the Approximate Number System, and the Cardinality Principle through Pearson correlations, multivariate regression, and mediation analysis with 5000 bootstrap samples.

**Results:**

Correlation analysis revealed that the Cardinality Principle was significantly and positively correlated with Inhibitory Control, Working Memory, Cognitive Flexibility, Executive Function, and the Approximate Number System. Regression analyses indicated that Executive Function positively predicted young children's Cardinality Principle. Specifically, Working Memory and Cognitive Flexibility were positive predictors of the Cardinality Principle, while Inhibitory Control was not. Mediation analysis results demonstrated that the Approximate Number System mediated the relationships between Inhibitory Control and the Cardinality Principle, Working Memory and the Cardinality Principle, and Cognitive Flexibility and the Cardinality Principle, respectively. In addition, the study found that young children's age negatively moderated the relationship between the Approximate Number System and the Cardinality Principle.

**Conclusions:**

The study emphasizes that in developing young children's Cardinality Principle, emphasis should be placed on improving their Executive Function and Approximate Number System while considering the age differences of young children and developing appropriate educational methods for different age groups.

## 1 Introduction

The Cardinality Principle (CP) refers to the understanding that the final number in a counting sequence represents the total number of items in the set (Paliwal and Baroody, 2020; Simon et al., 2023). This principle is fundamental for young children's acquisition of ordinal knowledge (Colomé and Noël, 2012), quantitative comparison (Abreu-Mendoza et al., 2013), spatial concepts (Berteletti et al., 2010), mathematical language (Hornburg et al., 2018), and early arithmetic skills (Paliwal and Baroody, 2020). It serves as a cornerstone for grasping formal mathematics and significantly influences young children's mathematical achievement in school (Paliwal and Baroody, 2020). Given its importance, the CP is pivotal to early mathematical cognition and forms the bedrock of effective math education.

However, the development of the CP is not an isolated process but rather one that intertwines with various cognitive factors. Despite extensive research, there is no consensus on the extent to which different cognitive abilities contribute to mathematics learning. Nonetheless, it is widely accepted that both domain-general and domain-specific abilities play crucial roles (Passolunghi et al., 2014). Research trends in recent years have shown that scholars are increasingly focusing on the key critical roles of Executive Function (EF, a domain-generalized ability) and Approximate Number System (ANS, a domain-specific ability) in CP formation and development (Odic et al., 2015; vanMarle et al., 2018; Sella et al., 2021; Bachman et al., 2022; Skagerlund et al., 2024).

EF is generally considered an umbrella term for higher-order cognitive processes necessary for the behavior of goal-directed individuals and is a domain-general ability. Its core elements includes inhibitory control, working memory, and cognitive flexibility (Diamond, 2013; Ganesan and Steinbeis, 2021). Inhibitory control involves the ability to suppress irrelevant information or inappropriate behavior to focus on a goal (Carlson and Wang, 2007). Working memory refers to the capacity to retain and manipulate task-related information during complex cognitive activities (Dunning and Holmes, 2014). Cognitive flexibility is the ability to adapt one's thinking and strategies in response to changing tasks or conditions (Highgate and Schenk, 2020). EF is critical for behavioral regulation, attention, and task-switching, and plays a foundational role in learning, particularly in mathematics (Bull and Lee, 2014; Zhang, 2016). While extensive research has demonstrated the importance of EF in early mathematical learning (Noël, 2009; Bull et al., 2011; Verdine et al., 2014; Mcclelland et al., 2014), most studies have focused on older children (Zhang, 2016). Few have explored how EF and its components affect the development of the CP in young children, leaving gaps in understanding the specific mechanisms involved.

The ANS is a domain-specific cognitive ability that enables individuals to make rapid, approximate quantitative judgments without relying on exact counting or symbolic numbers (Xu and Spelke, 2000). Cognitive neuroscience research indicates that the intraparietal sulcus, a key brain region, underpins the ANS (Dehaene et al., 1996). The ANS is considered crucial for developing CP, with empirical studies supporting this link (Halberda and Feigenson, 2008; Inglis and Gilmore, 2014). For instance, Fuhs et al. (2016) found a strong association between young children's ANS acuity and their development of the CP. vanMarle et al. (2018) also identified significant predictive relationships between the ANS and CP development. However, most research on this topic has been conducted outside China, and it remains unclear whether these findings apply to Chinese children.

Some scholars have pointed out that skills in general domains indirectly influence the development of mathematical competence through domain-specific skills (Östergren, 2013). This implies that from the theoretical level, there is a specific link between EF, ANS and CP and that ANS may be able to explain the relationship between EF and CP. Some scholars at home and abroad have conducted empirical studies on the relationship between EF, ANS and the development of the CP in young children. For example, a meta-analysis by Santana et al. (2022) covering 23 studies (mean = 69.6 months) found that higher cognitive flexibility contributes to improved comprehension of numbers. Ouyang et al. (2021) followed 138 young children (mean= 59.76 months) and showed a positive correlation between working memory and the accuracy of the ANS. Shusterman et al. (2016) studied 46 young children (mean= 50 months) between the ages of 36 and 60 months and showed that mastery of the CP time is synchronized with the development of their ANS. Not to be overlooked. The age of the young child is also one of the key factors. The association between young children's ANS and math ability has been found to decrease as they get older (Fazio et al., 2014; Peng et al., 2017; Schneider et al., 2017). This suggests that age may largely influence young children's link between the ANS and CP understanding, not only in terms of the existence of such a link but also in the strength of its effect. However, there are still gaps in our understanding of how EF affects CP, how ANS mediates this relationship, and the moderating role of age. In addition, the age of the subjects in previous studies has not been uniform, while some studies have shown that 4–7 years of age is an important period for the development of EF, ASN, and CP in young children (Jordan et al., 2006; Liu, 2013; Zhou, 2004). Therefore, the study used 4–7 young children as research subjects to explore whether ANS mediates the relationship between EF and CP and the moderating role of age, which can more effectively explore the relationship between young children's EF, ANS, and CP development, as well as provide educators with theoretical and practical insights in developing young children's mathematical skills.

### 1.1 Executive Function and young children's Cardinality Principle

The relationship between EF and mathematical abilities is well-documented, yet the specific link between EF and CP in young children remains less explored. Existing literature suggests that EF and the CP may share underlying physiological and cognitive processes. Research has identified brain regions such as the prefrontal cortex, orbitofrontal cortex, and anterior cingulate gyrus as critical for EF (Gao, 2009), while areas like the occipital-parietal-frontal regions are active during numerical processing (Thioux et al., 2001; Vandecruys et al., 2024).

In theoretical discussions, scholars have suggested that excellent inhibitory control is crucial for children when performing basic maths tasks such as counting and cardinal numbers. This ability helps children accurately recognize and count objects by following counting rules and effectively ignoring irrelevant distracting information (Fuhs and McNeil, 2013). In addition, the role of working memory cannot be underestimated, as it enables individuals to quickly and efficiently extract number facts and problem representations from long-term memory and to save the required counting information during problem-solving temporarily (Hubber et al., 2014). Further, cognitive flexibility is a key competency in children's mathematics learning, enabling them to switch flexibly between different mathematical strategies and concepts (Santana et al., 2022). As children progress from mere understanding of counting sequences to mastery of quantitative concepts, they must shift their thinking from a procedural to a conceptual level. This cognitive flexibility helps them better understand the quantitative information provided by counting (Purpura et al., 2017).

Empirical studies on EF and CP show mixed results. For instance, Scalise et al. (2021) found that inhibitory control predicted the CP, while Fuhs et al. (2016) did not find a significant correlation. Similarly, research on working memory has yielded conflicting findings, with some studies showing a positive correlation with the CP and others finding no significant relationship (Skagerlund et al., 2024; Goffin and Ansari, 2016). In some studies, cognitive flexibility has been linked to CP development, but the results are not universally consistent (Van der Ven et al., 2012; Fuhs et al., 2016; Santana et al., 2022). This lack of consensus highlights the need for further investigation into how different components of EF relate to the CP (Bull and Lee, 2014). Thus, this study hypothesizes:

***Hypothesis 1a (H1a)*****:** EF positively affects the CP.

***Hypothesis 1b (H1b)*****:** Inhibitory Control positively affects the CP.

***Hypothesis 1c (H1c)*****:** Working Memory positively affects the CP.

***Hypothesis 1d (H1d)*****:** Cognitive Flexibility positively affects the CP.

### 1.2 Executive Function and the Approximate Number System

The role of EF in ANS tasks has been explored from various perspectives. According to dual processing theory, cognitive processing involves both intuitive and analytical levels. Intuitive processing is rapid and unconscious, while analytical processing is deliberate and conscious (Evans and Stanovich, 2013; Keren, 2013). The ANS, which supports intuitive processing, allows for quick quantitative estimates without detailed counting. EF, linked to analytical processing, regulates and optimizes these intuitive estimates, ensuring their accuracy and flexibility (Evans, 2008).

Empirical studies have examined the relationship between EF and the ANS. Mueller and Brand (2018) found a negative correlation between errors in EF tasks and ANS performance, indicating that better EF is associated with higher ANS accuracy. Research focusing on sub-components of EF, such as inhibitory control, working memory and cognitive flexibility, has also explored their roles in ANS tasks. For instance, Fuhs and McNeil (2013) found that better inhibitory control was associated with higher ANS acuity. Similarly, Ouyang et al. (2021) showed a positive correlation between working memory and ANS accuracy. Cognitive flexibility has been linked to improved ANS performance, as demonstrated by Purpura and Simms (2018), who found significant correlations between cognitive flexibility and ANS accuracy.

### 1.3 Approximate Number System and the Cardinality Principle

Researchers have divided the human non-symbolic number system into the Exact Number System and the ANS. The ANS, present in both humans and animals, improves with age and is critical for understanding number concepts and developing mathematical skills (Halberda and Feigenson, 2008; Gilmore et al., 2011; Halberda et al., 2012). The intraparietal sulcus is crucial for number cognition and underpins the ANS (Dehaene et al., 1996). Studies have shown that the ANS is essential for developing the CP (Dehaene et al., 1998; Feigenson et al., 2004, 2013; Mussolin et al., 2016; Piazza, 2010). For example, Shusterman et al. (2016) found that the development of the CP coincided with ANS maturation, and vanMarle et al. (2018) identified significant predictive relationships between the ANS and the CP.

Contrary views exist, such as Schröder et al. (2022), who found no significant effect of ANS acuity at 1.5 years on later CP acquisition. Some researchers argue that the ANS and mathematical ability are independent (Carey, 2001, 2004; Rips et al., 2008; Butterworth, 2010; Libertus et al., 2014; Carey et al., 2017). Despite these conflicting views, most evidence supports a correlation between the ANS and mathematical ability. Empirical research on this topic is limited in China, and no studies have investigated the role of the ANS in mediating the relationship between EF and the CP. Thus, this study aims to examine this mediation effect in Chinese children.

***Hypothesis 2a (H2a):*** EF affects the CP through the ANS.

***Hypothesis 2b (H2b):*** Inhibitory Control affects the CP through the ANS.

***Hypothesis 2c (H2c):*** Working Memory affects the CP through the ANS.

***Hypothesis 2d (H2d):*** Cognitive Flexibility affects the CP through the ANS.

### 1.4 The moderating role of age

Recent findings suggest that age influences children's ANS acuity and CP. Specifically, as age increases, young children show a positive correlation in the acuity of ANS (Sekuler and Mierkiewicz, 1977; Holloway and Ansari, 2009). Zhou (2004) study noted that young children's understanding of CP was strongly correlated with their age. Hornburg et al. (2018) found that young children's age was significantly and positively correlated with their performance on the Give-n task but not with their performance on the how many tasks. Notably, recent studies have shown that the relationship between ANS and math ability diminishes with age (Fazio et al., 2014; Peng et al., 2017; Schneider et al., 2017; Inglis et al., 2011; Mussolin et al., 2012). For example, Inglis et al. (2011) found that young children's correctness and Weber coefficients on a quantity comparison task were significantly and positively correlated with scores on the Mathematics Achievement Test (Woodcock-Johnson III-Calculation). In contrast, this relationship was not significant in adults. A study by Mussolin et al. (2012) among preschoolers found that the correlation between ANS acuity and symbolic math ability was significant among 3- to 4-year-olds but not among 5- to 6-year-olds. However, this hypothesis was not supported by the results of Chen and Li (2014) meta-analysis, which found that the correlation between young children's ANS acuity and math proficiency did not change with age. This suggests that no academic consensus exists on the relationship between age, ANS acuity, and math ability. In addition, few studies have further explored whether age moderates the relationship between ANS acuity and young children's CP. Based on this, one of the goals of this study was to investigate whether age plays a moderating role in the relationship between ANS acuity and young children's CP.

***Hypothesis 3 (H3):*** Age negatively moderates the relationship between ANS acuity and young children's CP.

### 1.5 The present study

**Figure 1:**
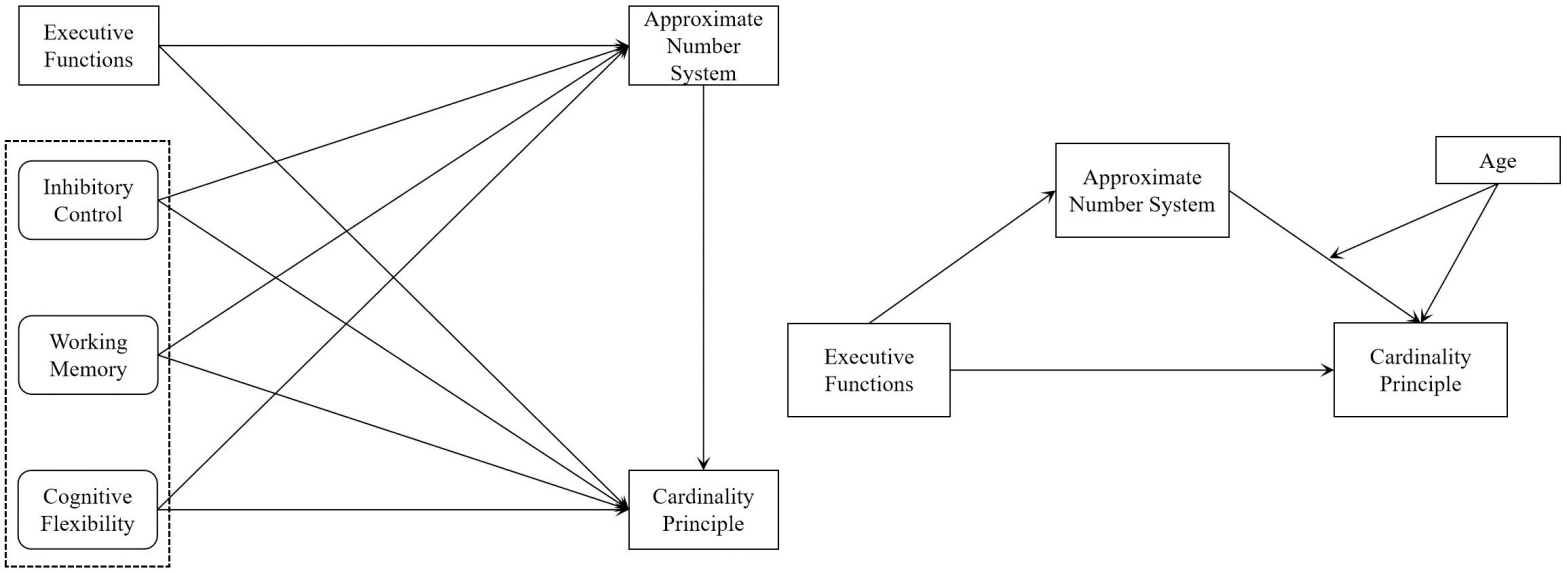
Research hypothesis model.

Research has explored the relationships between young children's EF, ANS, and the development of the CP. Studies have examined the connections between EF and ANS, EF and the CP, and ANS and the CP. These investigations have provided valuable insights into mathematical development in young children. However, several issues remain unresolved. First, the relationship between EF and CP is still debated, and there is a lack of research on the specific mechanisms through which EF influences the development of CP in young children. Second, while some studies have investigated how various sub-dimensions of EF—such as inhibitory control, working memory, and cognitive flexibility—affect the ANS, there is no consensus on these mechanisms. Lastly, there has been limited research on the interrelationships among young children's EF, ANS, and the development of the CP. In addition, few scholars have explored the moderating role of age in the relationship between ans and cp. To fill these gaps, the present study examined the mechanisms by which EF influences CP development, whether ANS mediates this relationship, and the moderating role of age. The following hypotheses were proposed in this study (see Figure 1).

***Hypothesis 1a (H1a)***: EF positively affects the CP.

***Hypothesis 1b (H1b)***: Inhibitory Control positively affects the CP.

***Hypothesis 1c (H1c)***: Working Memory positively affects the CP.

***Hypothesis 1d (H1d)***: Cognitive Flexibility positively affects the CP.

***Hypothesis 2a (H2a)***: EF affects the CP through the ANS.

***Hypothesis 2b (H2b)***: Inhibitory Control affects the CP through the ANS.

***Hypothesis 2c (H2c)***: Working Memory affects the CP through the ANS.

***Hypothesis 2d (H2d)***: Cognitive Flexibility affects the CP through the ANS.

***Hypothesis 3 (H3):*** Age negatively moderates the relationship between ANS acuity and young children's CP.

## 2 Method

### 2.1 Participants

Participants were recruited from preschools and kindergartens in Urumqi. Parental consent was obtained for 210 young children, but due to absences during testing, complete data were available for 203 young children. The sample consisted of 97 boys and 106 girls, aged 53–88 months (Mean age = 68.93 ± 7.076 months). Testing occurred in the young children's classrooms, and they received stickers or small prizes for their participation.

### 2.2 Measures

#### 2.2.1 Executive Function

EF was assessed through three sub-dimensions: inhibitory control, working memory, and cognitive flexibility. Based on this, the mean scores of the three sub-dimensions were summarized to become the young children's EF scores.

##### 2.2.1.1 Inhibitory control—Day-Night Stroop Task

Based on Marcovitch et al. (2015) Day-Night Stroop Task, young children were shown images of the sun and moon and instructed to say “night” when they saw the sun and “day” when they saw the moon. The task included 10 pictures of each image, with 20 formal tests following five practice trials. Scores ranged from 0 to 20 points. In this study, the Cronbach's α of this task is 0.948.

##### 2.2.1.2 Working memory—Digit Recall Task

Adapted from Gathercole et al. (2004), this task involved recalling sequences of digits read at a rate of one per second. The test started with sequences of 1 digit and increased in length until the child failed to recall three consecutive sets. The task consisted of seven lists ranging from 1 to 7 digits, with scores ranging from 0 to 21 points. In this study, the Cronbach's α of this task is 0.793.

##### 2.2.1.3 Dimensional Change Card Sort Task

Based on Zelazo (2006) Dimensionally Variable Card Categorization Task, young children sorted cards by color and shape. The test included six practice trials and 24 formal tests, with scores ranging from 0 to 24 points. The Cronbach's α for this task was 0.931.

#### 2.2.2 Approximate Number System

The ANS was evaluated using the Panamath test software developed by Halberda and Feigenson (2008), primarily using a dot-matrix comparison task. The task began with a “+” focus point on the computer screen, and when the “space” key was pressed, the left and right sides of the screen were provided with yellow dots and blue dots, respectively. The task requires the child to select the higher number of dots from the two dots and respond by pressing the appropriate key (labeled with the proper color on the keyboard beforehand). If the child failed to respond within the presentation time (2,128 milliseconds), the stimulus disappeared, a masking stimulus was presented, and a response box was presented with a key-press response to proceed to the next trial. The number of dots in each dot matrix ranged from 5 to 21, and the ratio of the number of yellow dots to blue dots ranged from 1.2 to 2.8 for random presentation. A total of eight practice trials and 80 formal trials were included, of which 40 trials had more yellow dots and another 40 had more blue dots. The 40 tests consisted of dots with the same number of dots and the same size of the total area of the dots, and the other 40 tests consisted of dots with the same number of dots and the same size of the total area of the dots. Test results were generated at the end of the test, i.e., the three metrics of accuracy, namely reaction time, accuracy and Weber's coefficient, were used as measures of ANS acuity (Peng et al., 2017; Inglis and Gilmore, 2014), and the test has been validated for use with Chinese children (Ouyang et al., 2021; Guo et al., 2021).

#### 2.2.3 Cardinality Principle

CP was assessed using the “Base Number Concept Measurement Task” and “Counting by Objects and Saying the Total Task” from Zhao (2006) and Zhang (2017). The mean scores of the two sub-tasks were summarized to become the young children's CP scores. The tasks involved:

##### 2.2.3.1 How Many Task

Young children were asked to count and report the number of blocks in sets of varying sizes, with scores ranging from 0 to 9 points. In this study, the Cronbach's α of this task is 0.846.

##### 2.2.3.2 Give-N Task

Young children were asked to provide a specific number of blocks from larger sets, with scores ranging from 0 to 9 points. In this study, the Cronbach's α of this task is 0.872.

### 2.3 Procedure

Ethical approval was obtained from the Shanghai Normal University Ethics Committee. Testing was conducted in schools with parental and school principal consent. Parents and teachers were informed that data would be used solely for research purposes and kept confidential. Trained researchers conducted the tests from May to June 2024. Each child participated in individual, one-on-one testing sessions lasting ~25–40 min.

### 2.4 Analysis

Data were analyzed using SPSS 29.0. Descriptive statistics were used to assess the current state of development in EF, ANS, and Cardinality. Correlation analyses examined relationships between these variables. Given that numerous studies have pointed out that gender and age affect EF, ANS, and CP (Sekuler and Mierkiewicz, 1977; Sabbagh et al., 2006; Holloway and Ansari, 2009; Acar, 2018; Hornburg et al., 2018), we tested the predictive power of EF and its subdimensions on CP by using gender and age as covariates. Mediation analyses using SPSS Process 4.1 tested whether the ANS mediated the relationships between EF sub-dimensions and the CP.

## 3 Results

### 3.1 Descriptive statistics and correlation

Descriptive statistics and Pearson's correlation coefficients are detailed in Table 1. The CP was significantly correlated with Inhibitory Control (*r* = 0.326, *p* < 0.001), Working Memory (*r* = 0.297, *p* < 0.001), Cognitive Flexibility (*r* = 0.426, *p* < 0.001), EF (*r* = 0.503, *p* < 0.001), and ANS (*r* = 0.575, *p* < 0.001). ANS was significantly correlated with Inhibitory Control (*r* = 0.318, *p* < 0.001), Working Memory (*r* = 0.233, *p* < 0.01), Cognitive Flexibility (*r* = 0.334, *p* < 0.001), and EF (*r* = 0.426, *p* < 0.001).

**Table 1:** Results of descriptive statistics and correlation analysis for each variable.

	***Mean***	***SD***	**1**	**2**	**3**	**4**	**5**	**6**
Inhibitory control	18.060	4.166	1					
Working memory	14.360	2.969	0.125	1				
Cognitive flexibility	21.500	4.388	0.375^***^	0.175^*^	1			
Executive Function	17.977	2.724	0.756^***^	0.521^***^	0.792^***^	1		
Approximate Number System	0.842	0.133	0.318^***^	0.233^**^	0.334^***^	0.426^***^	1	
Cardinality principle	6.862	2.285	0.326^***^	0.297^***^	0.426^***^	0.503^***^	0.575^***^	1

^*^p < 0.05, ^**^p < 0.01, ^***^p < 0.001.

### 3.2 Direct associations between Executive Function and young children's Cardinality Principle

After controlling for gender and age, linear regression analysis (Table 2) showed that EF (β = 0.394, *p* < 0.001) positively influences the CP, supporting Hypothesis 1a. Regarding sub-dimensions (see Table 3), Inhibitory Control (β = 0.112, *p* > 0.05) did not significantly impact the CP, refuting Hypothesis 1b. Working Memory (β = 0.179, *p* < 0.01) and Cognitive Flexibility (β = 0.272, *p* < 0.001) positively influenced the CP, supporting Hypotheses 1c and 1d.

**Table 2:** Regression analysis of the effect of Executive Function on Cardinality Principle.

**Predictors**		**Cardinality Principle**							
		***R** ^2^*	*Δ**R**^2^*	***F***	**B**	***SE***	β	***t***	**95% CI**
Model 1	Constant	0.229	0.221	29.727^***^	−4.362	1.47	–	−2.968	[−7.260, −1.464]
	Gender				0.511	0.283	0.112	1.801	[−0.048, 1.070]
	Age				0.152	0.020	0.469^***^	7.555	[0.112, 0.191]
Mode 2	Constant	0.369	0.36	38.855^***^	−1.41	1.409	–	−5.259	[−10.189, −4.632]
	Gender				0.281	0.259	0.062	1.082	[−0.231, 0.792]
	Age				0.115	0.019	0.355^***^	6.024	[0.077, 0.152]
	Executive function				0.331	0.050	0.394^***^	6.652	[0.233, 0.429]

^***^p < 0.001.

**Table 3:** Regression analysis of the effect of Executive Function's sub-dimensions on Cardinality Principle.

**Predictors**		**Cardinality Principle**							
		***R** ^2^*	*Δ**R**^2^*	***F***	**B**	***SE***	β	***t***	**95% CI**
Model 1	Constant	0.229	0.221	29.727^***^	−1.362	1.47	–	−2.968	[−7.260, −1.464]
	Gender				0.511	0.283	0.112	1.801	[−0.048, 1.070]
	Age				0.152	0.020	0.469^***^	7.555	[0.112, 0.191]
Model 2	Constant	0.378	0.362	23.963^***^	−1.683	1.426	–	−5.388	[−10.495, −4.871]
	Gender				0.296	0.259	0.065	1.141	[−0.216, 0.807]
	Age				0.116	0.019	0.358^***^	6.081	[0.078, 0.153]
	Inhibitory control				0.061	0.034	0.112	1.807	[−0.006, 0.128]
	Working memory				0.138	0.044	0.179^**^	3.108	[0.050, 0.225]
	Cognitive flexibility				0.142	0.032	0.272^***^	4.403	[0.078, 0.205]

^**^p < 0.01, ^***^p < 0.001.

### 3.3 The mediating role of the Approximate Number System

**Figure 2:**
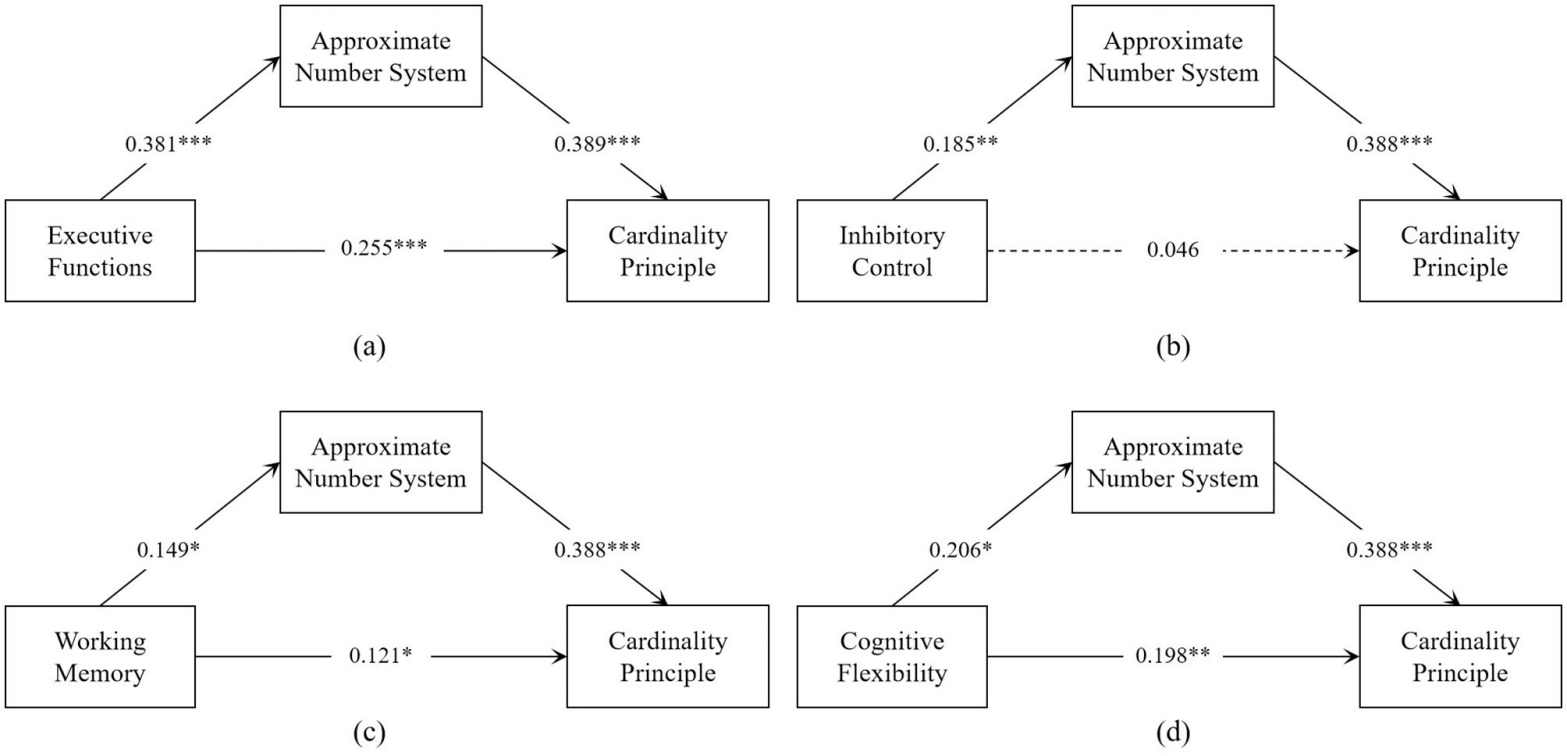
Approximate Number System mediated Executive Function and its sub-dimensions with Cardinality Principle. *p < 0.05, **p < 0.01, ***p < 0.001.

Since only one covariate, age, was significant in the linear regression, it was continued as a covariate in the mediation analysis. To more effectively explore the mediating role of ANS between the EF's sub-dimensions and CP, when mediation was analyzed for a sub-dimension, we added the other two sub-dimensions to the model as covariates. Mediation analysis (Tables 4, 5) revealed that EF was positively associated with the ANS (β = 0.381, *p* < 0.001), which in turn positively affected the CP (β = 0.389, *p* < 0.001). The residual direct effect was also significant (β = 0.255, *p* < 0.001), indicating that the ANS partially mediated the relationship between EF and the CP [indirect effect =0.148, bootstrap 95% CI (0.070–0.231)]. The mediating impact accounted for 36.725% of the total effect of EF on the CP. Inhibitory Control's effect on the CP was fully mediated by the ANS [indirect effect = 0.072, bootstrap 95% CI (0.006–0.145)], with the mediating impact accounting for 60.504% of the total effect. For Working Memory, the ANS partially mediated the relationship with the CP [indirect effect = 0.058, bootstrap 95% CI (0.006–0.113)], with the mediating impact accounting for 32.584% of the total effect. Cognitive Flexibility's effect was also partially mediated by the ANS [indirect effect = 0.080, bootstrap 95% CI (0.017–0.154)], with the mediating impact accounting for 28.777% of the total effect. The final path coefficients are illustrated in Figure 2.

**Table 4:** Analysis of the mediating role of the Approximate Number System between Executive Function and its sub-dimensions and the Cardinality Principle.

**Model**	**Predictors**	**ANS(M)**					**CP(Y)**				
		**B**	***SE***	***t***	β	**95% CI**	**B**	***SE***	***t***	β	**95% CI**
1	EF (X)	1.856	0.321	5.784	0.381^***^	[1.223, 2.488]	0.214	0.048	4.447	0.255^***^	[0.119, 0.309]
	ANS (M)	–	–	–	–	–	0.067	0.010	6.824	0.389^***^	[0.048 ~0.086]
		*R^2^* = 0.205, *F* = 25.777^***^					*R^2^* = 0.486, *F* = 62.713^***^				
2	IC (X)	0.591	0.221	2.670	0.185^**^	[0.155, 1.027]	0.025	0.031	0.814	0.046	[−0.036, 0.086]
	ANS (M)	–	–	–	–	–	0.067	0.010	6.823	0.388^***^	[0.047, 0.086]
		*R^2^* = 0.205, *F* = 12.772^***^					*R^2^* = 0.494, *F* = 38.421^***^				
3	WM (X)	0.665	0.29	2.289	0.149^*^	[0.092, 1.237]	0.093	0.040	2.29	0.121^*^	[0.013, 0.173]
	ANS (M)	–	–	–	–	–	0.067	0.010	6.823	0.388^***^	[0.047, 0.086]
		*R^2^* = 0.205, *F* = 12.772^***^					*R^2^* = 0.494, *F* = 38.421^***^				
4	CF (X)	0.623	0.21	2.963	0.206^**^	[0.208, 1.038]	0.103	0.030	3.489	0.198^**^	[0.045, 0.162]
	ANS (M)	–	–	–	–	–	0.067	0.010	6.823	0.388^***^	[0.047, 0.086]
		*R^2^* = 0.205, *F* = 12.772^***^					*R^2^* = 0.494, *F* = 38.421^***^				

IC, inhibitory control; WM, working memory; C, cognitive flexibility; ^*^p < 0.05, ^**^p < 0.01, ^***^p < 0.001.

All covariates of the mediating role were age.

**Table 5:** Analysis of the mediating effect of the Approximate Number System between Executive Function and its sub-dimensions and the Cardinality Principle.

	**Total effect**	**Indirect effect**	**Direct effect**	**The ratio of indirect to total effect**
Executive function	0.403 [0.241, 0.435]	0.148 [0.070, 0.231]	0.255 [0.119, 0.309]	36.725%
Inhibitory control	0.119 [−0.002, 0.131]	0.072 [0.006, 0.145]	0.046 [−0.036, 0.086]	60.504%
Working memory	0.178 [0.050, 0.225]	0.058 [0.006, 0.113]	0.121 [0.013, 0.173]	32.584%
Cognitive flexibility	0.278 [0.081, 0.208]	0.080 [0.017, 0.154]	0.198 [0.045, 0.162]	28.777%

The ratio of the indirect effect to the total effect is calculated as the proportion of the indirect effect relative to the total effect.

### 3.4 Mediation and moderated mediation models

**Figure 3:**
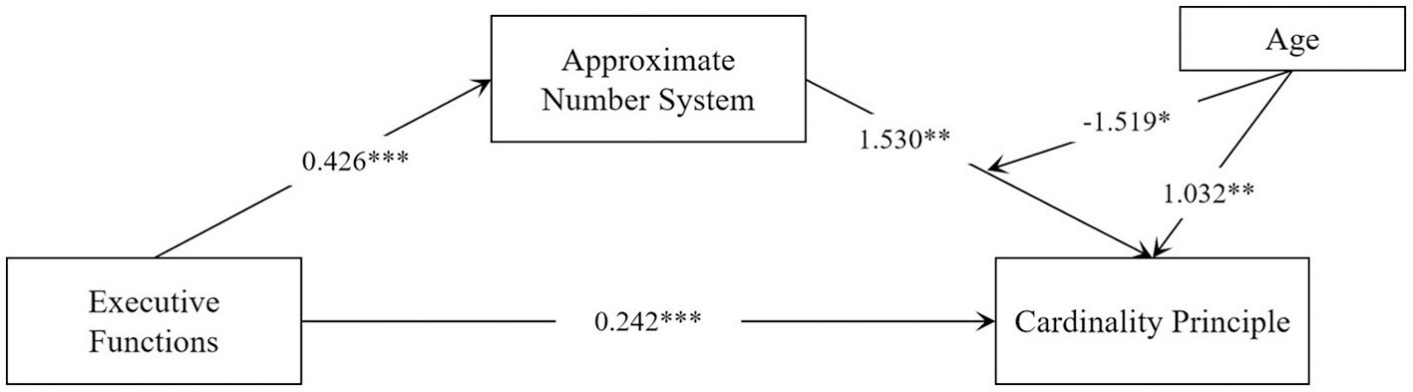
The moderating role of age as an Approximate Number System with Cardinality Principles. ^*^p < 0.05, ^**^p < 0.01, ^***^p < 0.001.

**Figure 4:**
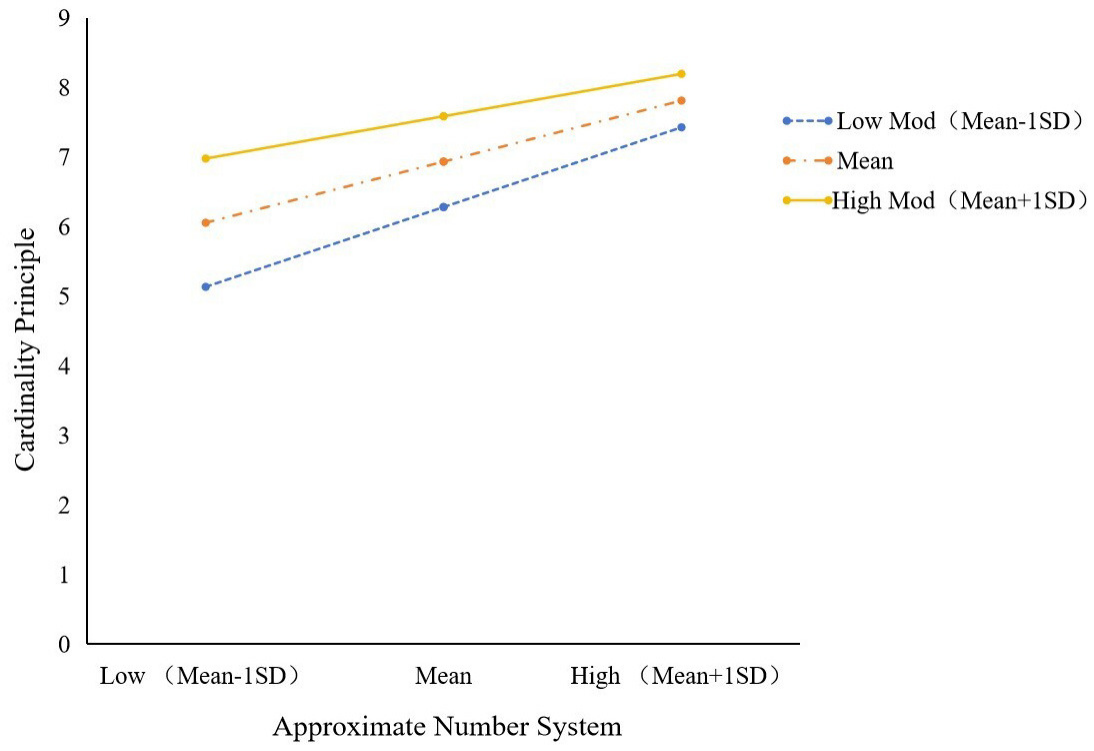
Indirect effects of Approximate Number System on Cardinality Principle under different age conditions.

To further investigate the relationship between the ANS and CP for young children in different age groups, the study analyzed the moderating role of young children's age in the mediator model by using the PROCESS macro program (model 14), and the results are shown in Table 6 and Figure 3. Both ANS (β = 1.530, *p* < 0.01) and young children's age (β = 1.032, *p* < 0.01) significantly and positively predicted cp, whereas the interaction term between ANS and young children's age (β = −1.519, *p* < 0.05) significantly and negatively predicted CP, suggesting that the young children's age plays a negatively moderating role in the relationship between ANS and CP. We then conducted a simple slope analysis to explore the nature of the moderating effect further (see Table 7; Figure 4). The results showed that in the low age level (mean – 1 SD), the effect was β = 0.086 [*p* < 0.001) (*p* < 0.001, 95% CI (0.062, 0.111)], and in the middle age level (mean), the effect was β = 0.066 [*p* < 0.001, 95% CI (0.047, 0.085)]. At high age level (mean + 1 SD), the effect was β =0.046 (*p* < 0.01, 95% CI (0.020–0.071)]. This suggests that the predictive effect of ANS on CP diminishes with age.

**Table 6:** Moderated mediation test.

**Variant**	**Cardinality principle**				
	**B**	***SE***	***t***	β	**95%CI**
Executive Function	0.203	0.048	4.258	0.242^***^	[0.109, 0.297]
Approximate Number System	0.263	0.081	3.244	1.530^**^	[0.103, 0.423]
Age	0.333	0.100	3.332	1.032^**^	[0.136, 0.530]
Approximate Number System^*^Age	−1.003	0.001	−1.437	−1.519^*^	[−0.005, −0.001]
*R^2^*	0.501				
*F*	49.687^***^				

^*^p < 0.05, ^**^p < 0.01, ^***^p < 0.001.

**Table 7:** Indirect effects of Approximate Number System on Cardinality Principle under different age conditions.

	**β**	**SE**	***t***	**95%CI**
Low age	0.086^***^	0.013	6.886	[0.062, 0.111]
Moderate age	0.066^***^	0.010	6.815	[0.047, 0.085]
High age	0.046^**^	0.013	3.527	[0.020, 0.071]

^**^p < 0.01, ^***^p < 0.001.

## 4 Discussion

Previous research has explored the relationships among young children's EF, ANS, and CP. However, there is a lack of consensus in the findings, and few studies have simultaneously examined the interactions among these three variables. This study aims to clarify the pathways through which EF influences the CP and to evaluate the mediating role of the ANS. Our findings yield two primary conclusions: (1) EF positively impacts the development of the CP in young children, (2) the ANS mediates the relationship between EF and the CP, and (3) Age Negatively Moderates the Relationship Between ANS and CP.

### 4.1 Executive Function and young children's Cardinality Principle

Consistent with previous studies (Purpura et al., 2017; Simanowski and Krajewski, 2019), our research confirms that EF positively predicts the CP. Young children with stronger EF exhibit a higher understanding of cardinality. Within EF, working memory and cognitive flexibility are found to be more significant predictors of CP compared to inhibitory control.

The lack of a significant direct correlation between inhibitory control and the CP may be attributed to the nature of the Day-Night Stroop Task used in this study. Gerstadt et al. (1994) noted that this task is challenging for younger children (3.5–4.5 years) but relatively easier for older children (6–7 years), which may reduce variability in inhibitory control among the participants. Additionally, young children's development of the CP may rely more on direct interactions with quantities and mathematical guidance rather than on inhibitory control alone (Levine et al., 2010). While inhibitory control plays a role in some mathematical tasks, it does not directly impact the CP as significantly as working memory and cognitive flexibility.

Empirical evidence supports that working memory and cognitive flexibility are more directly related to the CP (Fuhs et al., 2016; Skagerlund et al., 2024). Working memory is crucial for math learning as it involves retaining and manipulating numerical information. Early counting often relies on finger movements, which help young children track counted objects and reduce cognitive load (Kirsh and Maglio, 1994). Young children with robust working memory are better equipped to master the CP because they can manage and process numerical information more effectively.

Cognitive flexibility also plays a critical role by allowing young children to switch between different mathematical strategies and concepts (Santana et al., 2022). As young children progress from understanding counting sequences to grasping the concept of quantity, they must adapt their thinking from a procedural to a conceptual level. This flexibility enables them to better understand that counting provides quantitative information (Purpura et al., 2017), thus improving their mastery of the CP.

### 4.2 The mediating role of the Approximate Number System

Our study demonstrates that the ANS mediates the relationship between EF and CP. Specifically, the ANS mediates the effects of inhibitory control, working memory, and cognitive flexibility on the CP. As young children's EF components—such as inhibitory control, working memory, and cognitive flexibility—improve, their ANS acuity also increases, which in turn enhances their understanding of cardinality.

This finding aligns with Kang et al. (2020) theory. Inhibitory control positively influences the CP through its effect on the ANS. Young children with higher inhibitory control can better manage interferences during quantity estimation, allowing the ANS to focus more effectively on the quantity being estimated (Fuhs and McNeil, 2013). This enhanced focus improves the accuracy of estimation and provides a more reliable foundation for developing CP.

Similarly, working memory positively affects the CP by enhancing ANS acuity. Since working memory is essential for processing and controlling numerical information, improvements in working memory contribute to better ANS performance (Baddeley, 2018; Ouyang et al., 2021), which supports the development of CP.

Cognitive flexibility also impacts the CP through the ANS. In tasks requiring non-symbolic number comparison, young children with higher cognitive flexibility can effectively shift their focus and maintain the ANS's functionality (Kang et al., 2020). This flexibility aids in the formation and development of CP.

### 4.3 The moderating role of the age

The study showed that young children's age negatively moderated the relationship between ANS and CP. Specifically, the effect of ANS on CP gradually weakened as toddlers aged. This finding validated research hypothesis 3 and the theory previously proposed by Liang et al. (2021) that age may influence the strength of the relationship between ANS and math proficiency.

According to Piaget (1977) theory of stages of cognitive development, young children's cognitive abilities develop with age, and young children understand quantities primarily through visual and concrete information. At this time, their understanding of quantities may be based on ANS. As they grow older, children begin to use logic and more abstract thinking to understand quantities, reducing their reliance on ANS.

On the one hand, young children's symbolic quantity systems mature with age. Due to the symbolic quantity system's precision characteristics, young children's reliance on ANS in quantity processing gradually decreases, while their reliance on the symbolic quantity system gradually increases (Geary et al., 2013; Wong, 2020; Liang et al., 2021). On the other hand, early formal math education helps young children focus on quantitative attributes, increasing the quality and efficiency of their performance on ANS tasks, with a resulting greater effect on ANS (Shusterman et al., 2016).

## 5 Contributions, limitations, and future directions

This study reveals the relationship between EF, ANS and CP and explores the moderating role of age in this process. These findings have important implications for the practice of early childhood mathematics education. First, strengthening young children's EF training can help improve their understanding of CP. Educators should design rich and varied cognitive flexibility and working memory activities to promote the development of young children's abilities in these areas. Second, the mediating role of ANS between EF and CP suggests that improving young children's cognitive skills in non-symbolic quantities helps them better grasp mathematical concepts. Therefore, educational practices should focus on developing young children's intuitive perception of quantities and estimation skills. In addition, the negative moderating effect of age on the relationship between ANS and CP reminds us that educational strategies should be adjusted for young children of different ages. For younger children, educators should pay more attention to the cultivation of ANS ability and help them establish a basic understanding of quantity through concrete and intuitive mathematical activities. Older children should be gradually guided to transition from intuitive to abstract thinking, reduce their reliance on ANS, and strengthen the training of the symbolic quantity system. In conclusion, the results of this study show that in the process of mathematics education for young children, EF, ANS and age factors should be considered to develop a targeted educational program. At the same time, it provides useful references for parents and teachers to guide them on better assisting young children in learning mathematics daily to promote their all-round development.

Despite these contributions, the study has limitations. First, the cross-sectional design limits our ability to explore the temporal dynamics of these relationships. Future research should employ longitudinal designs to further investigate how these variables interact over time. Second, the study's focus on a specific age range of preschoolers may limit the generalizability of the findings to other age groups. Expanding the age range in future studies could provide a more comprehensive understanding of these developmental processes. Third, it has been shown that EF can mediate the relationship between ANS and young children's mathematical ability (Price and Wilkey, 2017; Lonnemann et al., 2019), but this was not taken into account in this study, and the relationship between the three can be further explored in the future by starting with the interactions between ANS and EF. Lastly, variables such as family socioeconomic status (SES) (Starkey et al., 2004; Mcneil et al., 2011; Ouyang et al., 2021) and vocabulary skills (Negen and Sarnecka, 2012), which might influence the ANS and the CP, were not included in this study. Future research should incorporate these additional variables to deepen the exploration of developmental mechanisms in mathematical cognition.

## 6 Conclusion

This study revealed the relationship between EF, ANS and CP in young children. The conclusions are as follows: first, EF has a positive effect on the development of CP in young children, with working memory and cognitive flexibility being more important predictors of CP. Second, ANS mediated the relationship between EF and CP, suggesting that EF promotes CP development by increasing the acuity of ANS. Finally, age negatively moderated the relationship between ANS and CP, with the effect of ANS on CP diminishing with age. This finding suggests that, in the process of young children's mathematics education, we should pay attention to the development of EF while focusing on the role of ANS and considering the influence of age factors on the development of mathematical cognition. Therefore, educators should design targeted teaching strategies according to young children's age characteristics and cognitive development level to promote the comprehensive development of young children's mathematical abilities.

## Data Availability

The data is not publicly available due to ethical requirements. Requests to access the datasets should be directed to the corresponding author or Huanhuan Li, huanhuanli@xjnu.edu.cn.
